# Fish, Mercury, Selenium and Cardiovascular Risk: Current Evidence and Unanswered Questions

**DOI:** 10.3390/ijerph6061894

**Published:** 2009-06-23

**Authors:** Dariush Mozaffarian

**Affiliations:** Division of Cardiovascular Medicine and Channing Laboratory, Brigham and Women’s Hospital and Harvard Medical School, and Departments of Epidemiology and Nutrition, Harvard School of Public Health, Boston, MA, USA; Tel.: +1-617-432-2887; Fax: +1-617-432-2435; E-Mail: dmozaffa@hsph.harvard.edu

**Keywords:** fish, mercury, selenium, cardiovascular disease, review

## Abstract

Controversy has arisen among the public and in the media regarding the health effects of fish intake in adults. Substantial evidence indicates that fish consumption reduces coronary heart disease mortality, the leading cause of death in developed and most developing nations. Conversely, concerns have grown regarding potential effects of exposure to mercury found in some fish. Seafood species are also rich in selenium, an essential trace element that may protect against both cardiovascular disease and toxic effects of mercury. Such protective effects would have direct implications for recommendations regarding optimal selenium intake and for assessing the potential impact of mercury exposure from fish intake in different populations. Because fish consumption appears to have important health benefits in adults, elucidating the relationships between fish intake, mercury and selenium exposure, and health risk is of considerable scientific and public health relevance. The evidence for health effects of fish consumption in adults is reviewed, focusing on the strength and consistency of evidence and relative magnitudes of effects of omega-3 fatty acids, mercury, and selenium. Given the preponderance of evidence, the focus is on cardiovascular effects, but other potential health effects, as well as potential effects of polychlorinated biphenyls and dioxins in fish, are also briefly reviewed. The relevant current unanswered questions and directions of further research are summarized.

## 1. Introduction

Controversy is present among the public and in the media regarding the health effects of fish consumption. Considerable evidence indicates that consumption of fatty fish reduces coronary heart disease (CHD) mortality [1] the leading cause of death in developed and most developing nations. On the other hand, concerns regarding potential harm from exposure to mercury [2–6], a heavy metal present in some fish species, have tempered the perception of fish as a healthy food. Very high levels of mercury exposure are known to cause sensorimotor symptoms in adults, which are often reversible when mercury exposure is reduced [7–9]. However, for the great majority of individuals, the main concern is the potential health effects of chronic low-level mercury exposure from modest fish consumption. Seafood species are also rich in selenium, an essential dietary trace element that plays an important role in antioxidant defense systems and may protect against both cardiovascular disease (CVD) and the toxic effects of mercury. A protective effect of selenium could directly inform recommendations relating to both optimal selenium intake and the potential impact of mercury exposure from fish consumption in selenium-replete vs. selenium-deficient populations. Because fish consumption appears to have significant health benefits, elucidating the relationship between fish intake, mercury exposure, and health risk is of considerable scientific and public health importance. The evidence for health effects of fish consumption in adults is reviewed, particularly the strength and consistency of evidence and relative magnitudes of cardiovascular effects of marine omega-3 polyunsaturated fatty acids (n-3 PUFA), mercury, and selenium in fish. Other potential health effects, including potential effects of polychlorinated biphenyls (PCBs) and dioxins in fish, are also briefly reviewed. This article does not consider possible cardiovascular benefits of plant-based omega-3 fatty acids, which have been reviewed elsewhere [10]. This review also focuses on health effects in adults – the potential effects on infant neurodevelopment, and corresponding recommendations for women who are or may become pregnant, have been reviewed elsewhere [1,11,12].

## 2. Fish and Cardiovascular Risk

**Figure 1.**
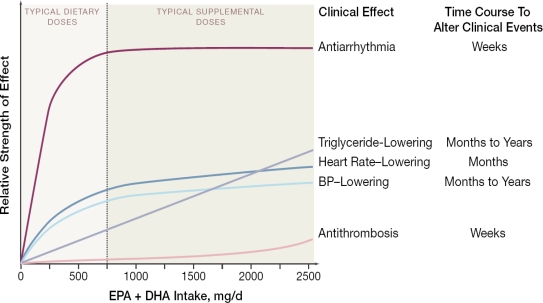
Schema of physiologic effects of n-3 PUFA consumption. The strength of effect denotes the relative impact of n-3 PUFA intake on clinical risk by means of the physiologic effect (e.g., triglyceride-lowering), whereas the time course to alter clinical events denotes the expected duration of intake for the benefits on the physiologic effect to be manifested in improved disease outcomes. For instance, the dose-response for anti-arrhythmic effects appears to be initially steep with a subsequent plateau, and effects on disease outcomes may be seen in weeks to months, whereas the dose-response for triglyceride-lowering is more gradual and monotonic, and effects on disease outcomes may require months to years of intake. Potentially important effects of n-3 PUFA on endothelial, autonomic, anti-inflammatory, and diastolic responses are not shown because dose- and time-responses of these effects are less well-established. Physiologic effects are not necessarily exclusive: e.g., anti-arrhythmic effects may be partly mediated by effects on blood pressure (BP) or heart rate. Reproduced with permission from Mozaffarian and Rimm [1].

Consumption of fish or fish oil favorably affects several cardiovascular risk factors (Figure 1) [13–24]. Changes in most risk factors are generally evident within weeks of changes in consumption and may result from altered cell membrane fluidity and receptor responses following incorporation of n-3 PUFA into cell membrane phospholipids [25,26] as well as direct binding of n-3 PUFA to cytosolic receptors that regulate gene transcription [27]. These physiologic effects of n-3 PUFA have varying dose-responses and time-responses of effect [1]. For example, at typical dietary intakes (< 1 g/d of n-3 PUFA), anti-arrhythmic effects appear to predominate, with such effects reducing the risk of cardiac death within weeks to months. At higher levels of consumption, maximum antiarrhythmic benefits appear to have been achieved, but now other physiologic effects of n-3 PUFA consumption (Figure 1) may begin to influence other clinical outcomes, such as stroke or nonfatal CVD events. Time courses of benefit also vary; for instance, some of these effects (such as triglyceride-lowering) might require months or years of consumption before an impact on incidence of clinical outcomes are evident. These benefits on intermediate risk factors are compelling, but inference for health effects of an exposure on chronic disease outcomes requires confirmation in studies of actual disease endpoints in humans.

**Figure 2.**
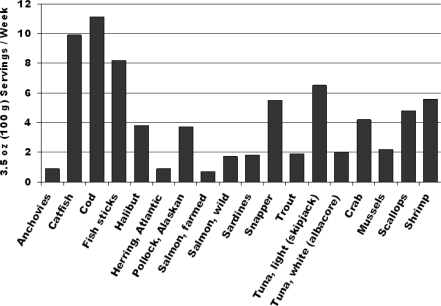
The number of 3.5 oz (100 g) fish servings per week needed to provide an average of 250 mg/day of the marine n-3 polyunsaturated fatty acids eicosapentaenoic acid (EPA) and docosahexaenoic acid (DHA). Based on data from Mozaffarian and Rimm [1].

This evidence exists. Results of case-control studies, prospective cohort studies, and randomized controlled trials each indicate that modest consumption of fish or fish oil lowers the risk of cardiac mortality, specifically CHD death and sudden cardiac death [1]. This effect appears to be nonlinear: compared with little or no intake, modest consumption (~250 mg/d) of the marine n-3 PUFA eicosapentaenoic acid (EPA) and docosahexaenoic acid (DHA) significantly lowers risk of cardiac mortality, whereas higher intakes do not substantially further lower risk, suggesting a threshold of effect [1]. The similarity of findings between observational studies of fish consumption and randomized controlled trials of n-3 PUFA supplementation suggests that, at least for cardiac mortality, much of the benefit of fish intake is related to the n-3 PUFA content. Consistent with this, when different types of fish meals are considered, lower risk is more strongly related to intake of fatty (oily or dark meat) fish, compared with lean (white meat) fish [28,29]. The quantity of fish servings needed to consume an average of 250 mg/d EPA+DHA varies depending on the particular fish species, but for fatty fish (e.g., anchovies, herring, salmon, sardines, trout, white tuna) is ~1–2 servings/week (Figure 2).

The strength and consistency of the evidence, and the magnitude of the benefit, for lowering of cardiac mortality by fish consumption are each notable. The risk reduction is supported by consistent evidence from experimental studies and randomized trials investigating effects of fish or fish oil consumption on cardiovascular risk factors; case-control studies evaluating fish consumption or objective biomarkers of n-3 PUFA intake and risk of cardiac outcomes; prospective studies of habitual fish consumption and cardiac outcomes that have followed hundreds of thousands of people for many years across a range of countries; and randomized controlled trials of fish or fish oil consumption that have enrolled thousands of subjects and demonstrated reductions in clinical events [1]. The magnitude of the benefit is also considerable: pooling of the prospective studies and controlled trials indicates that, compared with no intake, modest consumption (~250 mg/day [2¼ calories/day] of EPA+DHA, equivalent to ~1–2 servings/week of fatty fish) lowers risk of CHD mortality by 36% [1]. Analyses restricted to populations free of established heart diease (i.e., primary prevention) demonstrate similar results [30]. Fish intake may also reduce the risk of other cardiovascular and noncardiovascular outcomes, including but not limited to nonfatal heart attacks [31], ischemic stroke [32], atrial fibrillation [33], cognitive decline [34], and depression [35], but the evidence for these benefits is not yet as robust as for CHD death [1].

## 3. Mercury and Cardiovascular Risk

Mercury is a highly reactive heavy metal that is rarely found as a free element in Nature. In its elemental form, it is emitted from coal-burning electric power plants and used in chlorine production, dental amalgams, thermometers, and batteries [7,36]. Released into the air, it cycles from rain into streams, lakes, and oceans where it is converted by microorganisms into organic methylmercury (MeHg). Smaller amounts of inorganic mercury naturally in the environment (e.g., from volcanoes) may also be converted by these microorganisms to MeHg. When these microorganisms are ingested, MeHg bioaccumulates in the food chain from smaller creatures to larger predators, with tissue concentrations depending on the level of local contamination and on the size, lifespan, and predatory nature of each creature. Thus, MeHg levels tend to be higher in large, long-lived predators (e.g., shark, swordfish, king mackerel, tilefish); intermediate in medium-sized predators (e.g., trout, snapper); and lowest in short-lived (e.g., salmon) or smaller (e.g., shrimp, clams) species [1].

By strongly binding to sulfhydryl groups, mercury can alter the activity of a variety of enzymes, ion channels, and receptors [3,7]. In considering public health effects of chronic low-level exposure, the primary mercury species of interest is MeHg, which is more reactive and potentially toxic than elemental or inorganic mercury [7,36–38]. Elemental mercury is oxidized to mercuric ion, which does not readily cross some tissue barriers. Also, inorganic mercury is poorly absorbed in the gastrointestinal tract, limiting potential toxicity. In contrast, MeHg is readily absorbed in the gastrointestinal system and actively transported into tissues by a widely distributed amino acid carrier protein following the formation of a methylmercury-cysteine complex. Thus, compared with elemental or inorganic mercury, MeHg is more toxic at lower levels of exposure [7]. Additionally, with the exception of industrial accidents or occupational exposures to organic mercury, the major source of mercury exposure in humans is MeHg from fish [7,36,39,40]. Accordingly, the Mercury Study Report to Congress concluded, “Assessment of health endpoints, dose-response, and exposure suggests that methylmercury is the chemical species of major concern [7].”

Given their slow growth, mercury levels in toenails or hair provide the best biomarkers of chronic mercury exposure. Toenail mercury levels correlate with usual fish intake and, due to stability of most individuals’ dietary habits, are reproducible over time (r = 0.56 for toenail mercury samples obtained six years apart) [41,42]. This stability is similar to correlations of 0.6 – 0.7 typically observed, over a similar time interval, for widely used epidemiologic measures such as blood pressure [43]. Whereas both organic and inorganic mercury species contribute to total mercury levels, in the absence of unusual occupational or environmental exposures to inorganic mercury, MeHg from fish intake is the major determinant of variation in total mercury levels in hair and toenails. For example, the correlation between total mercury and MeHg levels in hair is 0.99 [44], and in toenails, this correlation is 0.97 (unpublished observation, in collaboration with Dr. Shade, Quicksilver Scientific, LLC, Lafayette, CO).

High exposure to mercury (often much higher than the U.S. reference dose) causes paresthesias, ataxia, and sensory symptoms in adults, which are often reversible when mercury exposure is reduced [7–9]. However, few individuals are exposed to such doses, and thus the major public health concern for the general population is the potential health effect of chronic low-level mercury exposure that could result from modest (up to several servings per week) fish consumption. For example, because such chronic low-level mercury exposure may have subtle effects on the developing brain in infants, the U.S. Food and Drug Administration and U.S. Environmental Protection Agency have issued specific recommendations regarding consumption of a few specific fish species to minimize mercury exposure in women who are or may become pregnant, nursing mothers, and young children [12,36].

Similar recommendations have not been released for the general population, because it is less clear that chronic low-level mercury exposure has significant health effects in adults. For example, outside of the sensitive period of brain development in the first years of life, current evidence is insufficient to conclude that chronic low-level mercury exposure has appreciable neurologic effects. As previously reviewed [1,11], in populations exposed to mercury from fish consumption, no clinical neurologic effects of mercury exposure are seen (excepting individuals with very high consumption, more than several servings per week, of fish highest in mercury [9]) and evidence for subclinical neurologic effects detectable with specialized testing is inconsistent [45–49]. Conversely, more consistent evidence suggests that fish consumption may favorably affect clinical neurologic events in adults, including ischemic stroke [32], cognitive decline and dementia [34], and depression and other neuropsychiatric disorders [35,50,51]. Thus, the balance of evidence does not suggest strong harm of fish consumption on neurologic outcomes in adults, but rather suggests significant potential benefits.

The most concerning potential health effects of chronic low-level mercury exposure in adults are on CVD risk and outcomes. *In vitro* and animal-experimental studies [2,3,6,52–61], as well as some observational studies of intermediate risk factors in humans [62–67], suggest that mercury has a variety of effects that could increase cardiovascular risk (Table 1).

While these experimental results and observational studies of intermediate risk factors are suggestive, investigation of actual disease endpoints in humans provides the best evidence to confirm potential effects of an exposure on chronic disease. Six studies have reported on the relations between mercury exposure and CVD endpoints in humans (Table 2) [68–73]. Among men in Kuopio, Finland, those in the highest third of hair mercury content (≥ 2.03 ug/g) had 66% higher risk of acute coronary syndromes compared to men in the lower third (< 0.84 ug/g) [72]. In two prospective studies in Sweden [68,69], higher blood mercury levels were not significantly associated with CVD risk. In a retrospective case-control study in Europe, men in the highest two quintiles of toenail mercury content (median levels 0.36 and 0.66 ug/g, respectively) had ~2-fold higher risk of nonfatal myocardial infarction, compared to men in the lowest fifth (0.11 ug/g) [70]. In a prospective study among U.S. men, toenail mercury concentrations were not significantly associated with CHD risk, even in the highest quintile (median level 1.34 ug/g) [71]. Finally, in a large prospective study in Sweden, erythrocyte mercury levels were not associated with risk of stroke [73].

Each of these studies had important potential limitations. In the Kuopio study, higher CHD risk was not seen until hair mercury levels exceeded ~2.0 ug/g, a level higher than the 95^th^ percentile of hair mercury levels among U.S. women of childbearing age (1.73 ug/g), nearly double the 90^th^ percentile (1.11 ug/g), and more than 10-fold higher than the average population exposure (0.19 ug/g). At hair mercury levels below ~2.0 ug/g in the Kuopio study, no significant relationships with CHD risk were seen; indeed, for CVD death and CHD death, individuals with hair mercury levels between 0.84 and 2.03 ug/g had trends toward *lower* risk than individuals with lower hair mercury levels. In the two earliest Swedish studies, relatively few events occurred, limiting statistical power. Ahlqwist *et al*. assessed mercury exposure in serum, which would reflect inorganic mercury from dental amalgams in addition to methylmercury from fish. Guallar *et al*. was a retrospective study in which only survivors of myocardial infarction were included; this could underestimate health benefits of fish consumption due to likely stronger benefits for fatal cardiac events (e.g., due to anti-arrhythmic effects), that were not included. In addition, more than one-third of eligible controls did not participate in this retrospective study, raising concerns for selection bias (i.e., the participating controls may not have been representative of the study base population giving rise to the cases). The U.S. cohort was large, prospective, and utilized toenail mercury, each of which would increase validity of results. Conversely, nearly 60 percent of participants were dentists, in whom mercury exposures would include both MeHg from fish consumption and inorganic mercury from working with mercury-containing dental amalgams [40]; thus, toenail mercury levels represented the combination of these exposures, which might have reduced the ability to detect associations with disease risk due to lower toxicity of inorganic mercury, compared with MeHg. When results were limited to the nondentists, trends toward higher CHD risk were seen with higher mercury levels, but results were not statistically significant due to fewer numbers of subjects in this subset [71].

**Figure 3.**
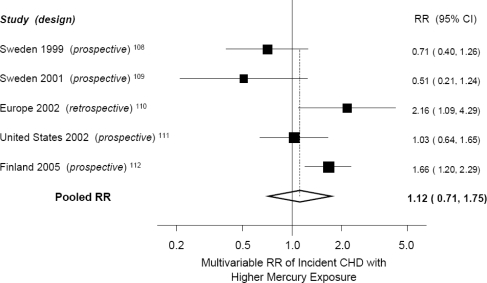
Meta-analysis of studies of mercury exposure and risk of coronary heart disease (CHD). Relative risk (▪) and 95% CIs (–) are shown comparing the highest to the lowest quantile of mercury exposure after adjustment for other risk factors. Adapted from Mozaffarian and Rimm [1].

Thus, the results of studies of mercury and cardiovascular events have been inconsistent, with only six published studies of this relationship and potential important limitations to each study. A meta-analysis of the five studies that evaluated CHD events (Figure 3) indicated no significant association between higher mercury exposure and risk of CHD (pooled RR = 1.12, 95% CI = 0.71–1.75) [1]. Most of these studies also excluded women, in whom CVD causes more deaths than the next seven causes of death combined, and did not evaluate stroke, the second leading cause of CVD morbidity and mortality [74]. Thus, the effect of mercury exposure on CVD risk has not been adequately studied, and U.S. regulatory agencies have identified this as a significant area of uncertainty requiring further attention [5]. Other quantitative analyses of risks and benefits of fish consumption also concluded that the current data is insufficient to quantitate the extent to which mercury affects CVD risk, and that the published evidence is also qualitatively ambiguous [75].

**Figure 4.**
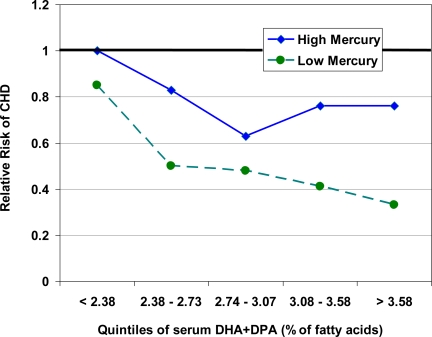
The interaction between exposure to n-3 PUFA and mercury from fish consumption. Serum docosahexaenoic acid (DHA) and docosopentaenoic acid (DPA) and hair mercury were measured in 1,871 participants who were prospectively followed for fatal or nonfatal acute coronary heart disease (CHD) [77]. Greater fish consumption (reflected by higher serum n-3 PUFA levels) was associated with lower CHD risk whether mercury exposure was high (> 2.0 ug/g, representing the highest tertile of exposure) or low (< 2.0 ug/g, representing the lowest two tertiles of exposure). However, the slope of this benefit was different: the higher relative risk of CHD (RR = 1.66) seen with higher mercury exposure in this study reflects, in essence, the difference in slope between these two lines. Thus, those with higher mercury exposure had less relative benefit - but not net harm – from higher fish consumption.

Notably, even if chronic mercury exposure were to increase CVD risk, the most important question for an individual decision regarding fish intake would be the tipping point of the relative harm from mercury vs. benefit from n-3 PUFA in the fish [76]. In other words, at what concentration of mercury, vs. content of n-3 PUFA, might the presence of MeHg change the health effects of fish consumption from net benefit to harm? Some data is available to help answer this question. First, in the two studies that observed higher cardiovascular risk with higher vs. lower mercury levels, the *net* relationship between overall fish consumption and CHD risk was still protective [70,72,77]. This suggests that, on average, fish consumption lowers CVD risk even in populations in which relative adverse effects of mercury were identified. Thus, the remaining uncertainty is: does this average beneficial effect of fish consumption differ at some level of mercury exposure? Unfortunately, most prior studies have not evaluated this potential interaction. The Kuopio investigators did report the interaction between effects of fish consumption, as reflected by serum n-3 PUFA levels, and effects of mercury exposure, as measured in hair [77]. Whether mercury levels were higher or lower, greater n-3 PUFA consumption was still associated with lower risk of CHD – higher mercury exposure simply lessened the slope of this benefit, but did not cause higher net risk (Figure 4). These findings suggest that, on average, (1) MeHg in fish may lessen the benefits of fish intake – a finding that has major implications for regulatory decisions regarding control of mercury emissions, because greater public health benefit may be derived from fish consumption if mercury levels were decreased; but (2) even consumption of mercury-containing fish provides some cardiovascular benefit compared with no fish consumption at all – a finding has major implications for an individual’s decision to consume or not consume a particular fish meal that contains mercury. Further investigation of this potential interaction, including both higher and lower ranges of both n-3 PUFA and mercury exposure, is clearly warranted.

## 4. Selenium and Cardiovascular Risk

Selenium, an essential dietary trace mineral, is a critical component of numerous selenoproteins in humans [78–80]. Food sources include fish and other seafood, red meat, eggs, chicken, and liver [81]. Wheat germ, brewer’s yeast, grains, and some vegetables may also be good sources, depending on the soil selenium concentration in which these crops were grown, that can vary substantially across different geographic regions [78,81]. Selenoproteins are important components of several antioxidant systems (e.g., glutathione peroxidase) that actively protect against damage from free radicals and reactive oxygen species [79,80]. Experimental studies suggest that these selenium systems may reduce CVD risk via several mechanisms (Table 3) [82–89]. For example, antioxidant defenses may reduce vascular and tissue injury resulting from formation of reactive oxygen species due to shear stress, hypoxia, hypertension, hyperlipidemia, and diabetes [82]. Selenium-related systems may also decrease the oxidation of lipids and protect the vascular endothelium from damage due to oxidized LDL cholesterol particles [83,84]. Selenium compounds may also more directly influence myocardial function and response to injury. In animal studies, selenium consumption increases cardiomyocyte glutathione peroxidase activity [85–87], improves cardiac recovery from ischemia-reperfusion injury [86], limits ischemia-induced structural alterations of mitochondria and sarcomeres [86], and reduces myocardial infarct size [87]. In diabetic animals, selenium improves cardiac mechanical and electrical dysfunction by restoring the cardiomyocyte glutathione redox cycle and restoring altered potassium currents [88]. Selenium also prevents myocyte structural abnormalities induced by diabetes, such as myofibril loss, reduction in myocyte diameter, and myofilament degeneration [89]. Selenium-intake also markedly reduces the incidence of ischemia-induced ventricular arrhythmias in rats [85].

As for n-3 PUFA and mercury, these experimental effects on intermediate risk factors require confirmation in studies of actual disease endpoints in humans. Variability of selenium content of foods from different regions renders selenium consumption difficult to estimate from dietary questionnaires, and thus measured biomarkers are optimal to allow valid and reproducible assessment of selenium intake. Selenium levels in blood or toenails provide reasonable ranking of differences in selenium consumption in a population [90]. For example, toenail selenium levels are higher with greater dietary selenium intake, assess average consumption over the prior 6 to 12 months, and are reproducible over time (r = 0.48 for toenail samples obtained six years apart) [42,90]. Toenail selenium levels are also higher among selenium supplement users, correlate in a dose-response fashion with supplement dose, and are consistent with the geographic distribution of selenium in forage crops [91].

Several studies have evaluated the relationship between selenium status and CVD endpoints, with inconsistent results. These studies have varied widely in terms of population studied, assessment of selenium exposure (e.g., blood, toenail, supplements), outcomes examined (e.g., CHD mortality, acute MI, combined CVD endpoints), and study size (ranging from 6 to 683 cases) [92]. In a prospective study among generally healthy U.S. men, no significant association was present between toenail selenium levels and total CHD events during five years follow-up (comparing extreme quintiles of selenium, the multivariate-adjusted RR was 0.86, 95% CI = 0.55–1.32, p trend = 0.75), but higher selenium levels were associated with a trend toward lower risk of nonfatal heart attacks (RR = 0.54, 95% CI = 0.31–0.93; p trend = 0.07) [93]. Retrospective case-control studies have generally seen stronger inverse associations between selenium levels and CVD risk than prospective studies that would be less susceptible to control selection bias. In addition to selection bias, possible limitations of some of these individual studies include low statistical power due to few numbers of events, or evaluation of combined endpoints, e.g., including elective coronary revascularization procedures as outcomes. A meta-analysis of 25 observational studies that measured blood or toenail selenium concentrations and six randomized trials that evaluated selenium supplements demonstrated a pooled relative risk for CHD of 0.43 (95% CI = 0.29–0.66) in case-control studies and 0.85 (95% CI = 0.74–0.99) in cohort studies, comparing the highest with the lowest selenium concentration categories, and 0.89 (95% CI = 0.68–1.17) in randomized trials, comparing supplement users to nonusers [92]. The effect of selenium consumption on CVD risk may also be modified by duration of consumption, smoking, alcohol use, or antioxidant vitamin intake, the influences of which were not evaluated in most prior studies. Thus, while animal-experiments suggest that selenium may protect against CVD, an American Heart Association Science Advisory in 1999 concluded that “little information is available on the preventive effects of [selenium] in human populations [94].”

Selenium intake is thought to be adequate in many populations [95], which may limit detection of relations between selenium intake and disease risk. However, the threshold for adequate selenium intake is based on prevention of Keshan disease (a rare cardiomyopathy) and on secondary functional measures (such as glutathione peroxidase activity) [96]. The threshold - if any - for prevention of CVD is unknown. As described above, in one study among U.S. men, selenium levels were associated with a trend toward lower risk of nonfatal heart attacks [93], suggesting that selenium may influence cardiovascular risk even in populations with supposedly adequate intake. Nevertheless, overall, the associations of selenium intake with CVD risk, nor any potential thresholds of effect, are currently not well-established.

## 5. Interaction between Mercury, Selenium and Cardiovascular Risk

While the relations of mercury and selenium with cardiovascular risk are controversial, perhaps the most interesting possibility is that these relations are not independent. It has long been hypothesized that selenium may protect against toxic effects of mercury, particularly organic MeHg [97–102]. Evidence from both *in vitro* studies and experiments in animals supports this concept [97–110]. Selenium protects against mercury toxicity in rats [100], mice [105], and quail [103], and against mercury-induced apoptosis in human cell lines [108]. Cardiovascular protection may be related to antioxidant effects of selenoprotein systems, which could minimize damage from mercury-induced free radicals [100]. In rats, the mercury-induced reductions in plasma and liver glutathione S-transferase activity can be alleviated by co-administration of selenium [107]. Such protection could also result from direct effects of selenium on mercury kinetics and metabolism. Reduced selenite (for example, from glutathione peroxidase activity) degrades MeHg to less toxic inorganic mercury [104]. Additionally, glutathione-reduced selenite (selenide) binds with mercury and plasma selenoprotein P to form a ternary complex [109], although the biologic significance of this is unclear. Interestingly, in a small randomized trial among human subjects with low selenium intake (n = 23), selenium supplementation reduced hair mercury levels by one-third over four months [110].

Epidemiologic studies indirectly support a possible protective effect of selenium intake on cardiovascular toxicity of mercury [111]. Mercury exposure has been associated with cardiovascular risk in European populations with possibly low selenium intake [70,72], but not in a U.S. population with likely higher selenium intake [71]. However, in the only prospective study that directly evaluated this potential interaction, selenium levels did not significantly modify relations between mercury and CHD events [71], although statistical power may have been inadequate to detect an interaction.

Thus, *in vitro* studies and animal-experiments suggest that selenium may protect against mercury toxicity, but it is unclear whether these findings are relevant to selenium intake and mercury exposure in humans. Understanding whether selenium intake prevents the potential CVD toxicity of mercury is important both for determining the appropriate level of public health concern for mercury exposure from fish consumption in different populations and for making recommendations regarding optimal selenium intake in the U.S. and other countries. Unfortunately, the possible interaction between mercury, selenium, and cardiovascular risk has not been studied adequately to draw any firm conclusions.

## 6. Polychlorinated Biphenyls (PCBs) and Dioxins

**Figure 5.**
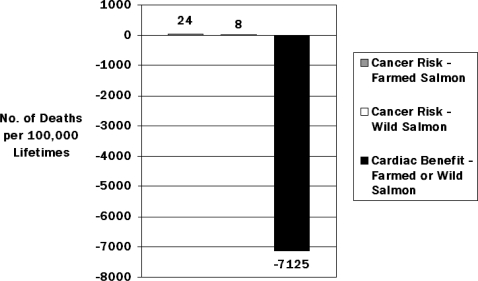
Calculated cancer risks vs. cardiac benefits from a lifetime of regular consumption of farmed or wild salmon, based on contents of PCBs/dioxins and n-3 PUFA in farmed and wild salmon [114]. The estimated cancer risks - 24 vs. 8 deaths for farmed vs. wild salmon, respectively – are orders of magnitude smaller than the calculated cardiac benefits of 7,125 fewer cardiac deaths for either farmed or wild salmon consumption.

Although the focus of this review is on n-3 PUFA, mercury, and selenium, the potential health effects of PCBs and dioxins present in some fish species merit consideration. Exposure to these contaminants has been declining steadily since the 1970s [112], when changes in regulatory policies and industry practices reduced production and emission levels, lowering levels in foods. Nevertheless, they persist in the environment and human tissues for a long time, and attention on their levels in fish species was rekindled following publication of a comprehensive analysis that farmed salmon contained higher levels of PCBs/dioxins than wild salmon [113], a result that received considerable media attention. Placed in perspective, however, the average levels in farmed salmon were low, only ~2% of the FDA action level. Thus, the main finding was not that average levels of PCBs/dioxins in farmed salmon were high, but simply that average levels in wild salmon were even lower. Most importantly, when the potential cancer risks of the PCBs/dioxins in both farmed and wild salmon were quantitatively compared to the expected CHD benefits from the n-3 PUFA, the estimated magnitudes of the hypothesized cancer risk were orders of magnitude lower than the reduction in cardiac mortality (Figure 5) [114]. Three additional points deserve attention. First, the estimates of cancer risk were based on 70-year lifetimes of eating salmon at levels to achieve 1000 mg/d EPA+DHA, or approximately seven 3.5-oz wild salmon servings/week or three 3.5-oz farmed salmon servings/week for 70 years. However, based on the nonlinear relationship between EPA+DHA intake and CHD mortality [1], a similar CHD mortality reduction would likely occur at one-fourth of the consumption level (i.e., 250 mg/d EPA+DHA), but with only one-fourth of the cancer risk (i.e., 6 or 2 excess cancer deaths per 100,000 lifetimes for farmed or wild salmon, respectively). Second, the estimated cancer risks of PCBs/dioxins include a 10-fold uncertainty factor; i.e., the true effects at these consumption levels may be 1/10 as large, or 0.6 or 0.2 excess cancer deaths per 100,000 lifetimes for farmed or wild salmon, respectively. Finally, the estimated cancer risks of PCBs/dioxins are largely derived from extrapolation of results from limited high human exposures and animal-experiments, whereas the calculated benefits for CHD death are derived from multiple large prospective cohort studies and randomized controlled clinical trials in humans of fish and n-3 PUFA consumption and actual CHD mortality [1,114]. Thus, whether the true excess cancer risk for 70-years consumption of farmed or wild salmon would be 24 or 8, 6 or 2, or 0.6 or 0.2 deaths per 100,000 lifetimes, these risks pale in comparison, both in magnitude and strength of evidence, to the many thousands of CHD deaths that would be prevented by such consumption. Because levels of PCBs/dioxins may be higher in some freshwater fish species caught in certain local rivers or lakes, sports fishermen who regularly consume locally caught freshwater fish should consult local advisories. However, for commercially purchased fish and shellfish, based on the relatively low contents of PCBs/dioxins [1], the greater strength and quality of evidence for CHD benefits of n-3 PUFA vs. cancer risks of PCBs/dioxins, and the quantitatively far larger CHD benefits compared with the hypothesized cancer risks, the great majority of individuals who consume commercially purchased fish should not be concerned about PCB or dioxin content in making fish consumption decisions; indeed, such concern may substantially increase health risk by leading to reduced fish consumption. Continued attention to reducing PCB and dioxin emissions into the environment is important, but in contrast to MeHg exposure, this is not a seafood-specific issue: the majority (> 90%) of PCBs and dioxins in the food supply are consumed from non-seafood sources, in particular meats, dairy products, and vegetables [115,116].

## 7. Conclusions

Consistent evidence from human experimental studies, case-control studies, prospective cohort studies, and randomized controlled trials indicates that modest consumption of fish significantly reduces cardiac death. The magnitude of benefit is substantial, with 36% lower risk with consumption of 250 mg/d EPA+DHA (equivalent to ~1–2 servings of fatty fish per week), compared with no intake. The strength, quality, and consistency of evidence for health effects of chronic low-level mercury exposure in adults are much less robust, indicating a need for further investigation of possible effects of such exposure on disease outcomes in appropriately powered prospective human studies. The conflicting results of prior studies of mercury and CVD risk are not dose-related, as some studies with relatively low exposures observed a positive association, whereas other studies found no significant associations at similar or higher exposures. Notably, even in the two studies that observed a positive relationship between mercury exposure and cardiovascular risk, the overall average effect of fish consumption was protective: mercury exposure appeared to lessen, but not reverse, the cardiovascular protection of fish intake. Animal-experiments suggest that selenium may protect against CVD, but studies of selenium intake and cardiovascular endpoints in humans are inconsistent, and a possible interaction between mercury and selenium for CVD risk has not been adequately studied in humans. Based on the current evidence, the health risks for adults of not consuming fish outweigh potential risks from mercury or other contaminants. Modest consumption (1–2 fish servings per week) appears to provide most of the reduction in CHD mortality, and concerns over possible accompanying effects of mercury or other contaminants in adults can be curtailed by simply choosing a variety of different fish and seafood [1,11].

Several unanswered questions remain. First, growing evidence suggests that fish consumption has benefits for health outcomes beyond CHD mortality, including nonfatal cardiovascular events and several noncardiovascular outcomes: these potential effects, and their dose-responses, require further investigation. Second, whereas current evidence does not support a need for any advisory to limit intake of fish or specific fish species for health effects in adults, further research is needed to elucidate the potential health effects of mercury exposure and the level, if any, at which potential effects of mercury could equal or outweigh the benefits from n-3 PUFA consumption. Even if further research ultimately confirms that, as suggested by current evidence, the net health effect of any single fish meal is nearly always beneficial, the extent to which mercury might reduce this net benefit must be evaluated to inform regulatory decisions regarding control of mercury emissions, in that greater public health benefit may be derived from fish consumption if mercury levels were decreased further. Third, whether selenium protects against CVD risk and/or against potential toxic effects of mercury, as well as any threshold of such effects, must be better established. Finally, although not a focus of this present review, both the intended and unanticipated consequences of government advisories, public health recommendations, and media reports regarding health effects of fish consumption must be better understood, so that well-intentioned messages do not cause confusion or ultimate harm. The answers to these questions will have significant implications for our understanding of how n-3 PUFA, mercury, and selenium influence health risk and will clarify the most appropriate emphases for recommendations regarding fish intake to improve health in adults. As future studies are completed, continued careful consideration of the strength and consistency of the evidence, as well as the relative magnitudes of effect, is imperative.

## Figures and Tables

**Table 1.** Experimentally-observed effects of mercury which may increase CVD risk.

**Systemic Effects**
Promotion of free radicals and reactive oxygen species
Inhibition of antioxidant systems (glutathione peroxidase, catalase, superoxide dismutase)
Increased lipid (e.g., LDL cholesterol) peroxidation
Promotion of blood coagulation (clotting)
Inhibition of endothelial cell migration
**Direct Cardiovascular Effects**
Reduction in myocardial contractile force
Increased calcium release from myocardial sarcoplasmic reticulum
Reduction in left ventricular myosin ATPase activity
Decreased heart rate variability and increased blood pressure

**Table 2.** Prior studies of mercury and cardiovascular events in humans.

**Author, Year**	**Design**	**Country**	**Exposure**	**Outcome**	**No. of Cases**	Relative Risk (5% CI) with High Mercury *
Ahlqwist, 1999	Prospective cohort	Sweden	Serum mercury	Total myocardial infarction, Stroke	87, 77	RR = 0.71 (0.40, 1.26)† for MI; RR = NS (data not reported) for stroke
Hallgren, 2001	Prospective (nested) case-control	Sweden	Erythrocyte mercury	Total myocardial infarction	78	RR = 0.51 (0.21, 1.24)
Guallar, 2002	Retrospective case-control	8 European countries and Israel	Toenail mercury	Nonfatal myocardial infarction	684	RR = 2.16 (1.09, 4.29)
Yoshizawa, 2002	Prospective (nested) case-control	USA	Toenail mercury	Total myocardial infarction + coronary revascularization	470	RR = 1.03 (0.65, 1.65)
Virtanen, 2005	Prospective cohort	Finland	Hair mercury	Total acute coronary events	282	RR = 1.66 (1.20, 2.29)
Wennberg, 2007	Prospective (nested) case-control	Sweden	Erythrocyte mercury	Stroke	369	RR = 0.99 (0.93, 1.06) in men; RR = 1.00 (0.94, 1.08) in women

* Multivariable-adjusted relative risk (RR) comparing the highest vs. the lowest category of mercury levels, except for Wennberg *et al*. for which the RR corresponds to each one ng/g increase in erythrocyte mercury.

† Personal communication (Calle Bengtsson, June 6, 2006). NS = nonsignificant.

**Table 3.** Experimentally-observed effects of selenium which may reduce CVD risk.

**Systemic Effects**
Antioxidant defense against free radicals and reactive oxygen species
Decreased lipid peroxidation
Protection against vascular damage from oxidized LDL cholesterol particles
Antithrombotic effects from decreased plasma thromboxane A2
**Direct Cardiovascular Effects**
Increased myocardial antioxidant glutathione peroxidase activity
Improved cardiac recovery from ischemia-reperfusion injury
Limitation of ischemia-induced and diabetes-induced ultrastructural damage
Reduction in myocardial infarct size
Restoration of altered myocyte ion currents
Reduced incidence of ischemia-induced ventricular arrhythmias
